# Utility of Thermographic Imaging for Callus Identification in Wound and Foot Care

**DOI:** 10.3390/s23239376

**Published:** 2023-11-23

**Authors:** Faraz Sadrzadeh-Afsharazar, Rose Raizman, Gennadi Saiko

**Affiliations:** 1 Department of Electrical, Computer and Biomedical Engineering, Toronto Metropolitan University, Toronto, ON M5B 2K3, Canada; 2 Lawrence S. Bloomberg Faculty of Nursing, University of Toronto, Toronto, ON M5S 1A8, Canada; 3 Scarborough Health Network, Toronto, ON M1E 4B9, Canada; 4 Department of Physics, Toronto Metropolitan University, Toronto, ON M5B 2K3, Canada

**Keywords:** wound care, foot care, callus, debridement, clinical thermography

## Abstract

Calluses are thickened skin areas that develop due to repeated friction, pressure, or other types of irritation. While calluses are usually harmless and formed as a protective surface, they can lead to skin ulceration or infection if left untreated. As calluses are often not clearly visible to the patients, and some areas of dead skin can be missed during debridement, accessory tools can be useful in assessment and follow-up. The practical question addressed in this article is whether or not thermal imaging adds value to callus assessment. We have performed a theoretical analysis of the feasibility of thermographic imaging for callus identification. Our analytical calculations show that the temperature drop in the epidermis should be on the order of 0.1 °C for the normal epidermis in hairy skin, 0.9 °C for glabrous skin, and 1.5–2 °C or higher in calluses. We have validated our predictions on gelatin phantoms and demonstrated the feasibility of thermographic imaging for callus identification in two clinical case series. Our experimental results are in agreement with theoretical predictions and support the notion that local skin temperature variations can indicate epidermis thickness variations, which can be used for callus identification. In particular, a surface temperature drop on the order of 0.5 °C or more can be indicative of callus presence, particularly in callus-prone areas. In addition, our analytical calculations and phantom experiments show the importance of ambient temperature measurements during thermographic assessments.

## 1. Introduction

Corns and calluses are thickened skin areas that develop due to repeated friction, pressure, or other types of irritation. They are formed by accumulating terminally undifferentiated keratinocytes in the outermost layer of skin. Although the cells of calluses are dead, they are highly resistant to mechanical and chemical damage, due to extensive networks of cross-linked proteins and hydrophobic keratin intermediate filaments that contain many disulfide bonds [1]. Calluses are a natural response to skin irritation on the palms or soles of the feet. However, excessive friction that occurs too quickly for the skin to develop a protective callus can cause blisters or abrasions instead.

While there is some ambiguity in the literature on the exact definitions of corns and calluses, we will use a definition by Singh et al. [2]: “A callus is a broad-based, diffuse area of hyperkeratosis of relatively even thickness, most commonly found under the metatarsal heads. A callus is less circumscribed than a corn, is usually larger, does not have a central core, and may or may not be painful.”

Corns and calluses are mechanically induced hyperkeratosis [3]. The formation of a callus is a complex process that involves several biochemical and mechanical changes in the skin. Mechanical stress activates keratinocytes in the epidermis, which begin to divide and differentiate faster. This leads to the formation of a thicker layer of keratin, the protein that makes up the stratum corneum. Mechanical stress also causes the accumulation of extracellular matrix molecules such as collagen and glycosaminoglycans, which contribute to the thickening of the stratum corneum.

Several risk factors, such as foot deformities (e.g., bunions, hammertoe) and not wearing socks or protective gloves, can contribute to the formation of calluses. While calluses are usually harmless, if left untreated, they can lead to ulceration or infection, as hyperkeratinization may result in the breakdown of skin and tissue integrity. In particular, callus is a major risk factor in ulcer development (a relative risk of 11.0 [4]), which is particularly important for patients with diabetes. Calluses can also cause patients to shift their weight off the affected, painful area, placing excessive stress on the asymptomatic side.

Therefore, removing calluses is essential to treating and preventing diabetic foot ulcerations (DFU). However, patients often have difficulty identifying calluses, which are located on the plantar foot, often unsightly, and not as clearly visible as corns. As a result, some areas of dead skin can be missed during the debridement process. Thus, a tool that can identify such areas could be clinically important.

Several approaches have been proposed to assist with callus identification, including diffuse reflectance techniques [5] and water content imaging [6]. However, these imaging technologies are at an early stage and have not yet been clinically adopted. Thus, building a clinical tool based on a proven technology can help to accelerate adoption.

Thermography has significant potential as an adjuvant technique in diabetic foot care. Under controlled conditions, skin temperature directly reflects arterial blood flow, which transports oxygen, nutrients, and heat to the extremities [7]. For example, elevated temperature is a reliable marker of inflammation and can thus predict the risk of ulceration, infection, and amputation [8]. Similarly, decreased temperature may be a sign of insufficient blood supply and indicate ischemia [9].

The utility of thermography in wound care has been known since the early 1960s when Lawson et al. [10] used infrared scanning to predict burn depth with an accuracy of 90%, as confirmed via histology. However, for many years, the technology was confined to research laboratories. With recent advances in the development of inexpensive microbolometers, thermography started gradually penetrating routine clinical practice.

In [11], it was suggested that callus can be identified using thermography. An analytical model predicted that the epidermis temperature drop, Δ*T_e_*, is relatively small (0.1 °C) for most body parts (non-glabrous skin). However, it can be on the order of 0.5–1 °C for glabrous skin and 1.5–2 °C or even higher for calluses. This finding can have practical implications in wound care and foot care for callus identification. If this prediction is correct and we observe the skin temperature in two nearby areas, the area with callus will have a temperature of >1 °C lower than nearby callus-free areas. The practical question is how to identify the callus areas by looking at thermal images.

For this reason, we aimed to verify experimentally and subsequently clinically assess the feasibility of callus identification using thermal imaging. For this purpose, we performed a theoretical analysis and validated our predictions on a gelatin phantom. Since the purpose of this study is mostly proof-of-concept, we limited our clinical experiments to two case series.

## 2. Materials and Methods

### 2.1. Analytical Model

Here we consider the simple analytical model of heat transfer in the skin developed in [11], with a particular focus on the epidermis. Heat is generated (delivered mainly by warm arterial blood) in the dermis, transferred through the epidermal layer, and dissipated in the ambient air. More specifically, heat transfer in human skin can be modeled using the bioheat equation by Pennes [12],

(1) $$\rho C\frac{𝜕T}{𝜕t}=k\nabla^{2}T+\rho_{b}C_{b}\omega_{b}\left(T_{b}-T\right)+Q$$

where *ρ*, *C*, *T*, *k*, and *Q* denote the tissue density (kg/m^3^), the specific heat of the tissue (J/kg∙K), the local tissue temperature (K), the thermal conductivity of the tissue (W/m∙K), and metabolic heat generation per unit volume (W/m^3^), respectively. *ρ_b_*, *C_b_*, *T_b_*, and *ω_b_* represent the blood density (kg/m^3^), the specific heat of the blood (J/kg∙K), the arterial blood temperature (K), and the blood perfusion rate (m^3^/s/m^3^), respectively. 

Heat loss from human skin to the ambient air [W/m^2^] can be described as

(2) $$q=h\left(T_{skin}-T_{\infty }\right)$$

where $T_{\infty }$ is the ambient air temperature, and *h* is a heat transfer coefficient (*h =* 12 W/m^2^K, [13]).

We can consider the epidermis to be a slab of nonviable tissue with thickness *E*. The effect of metabolic heat generation was considered in [11] and was found quite negligible, even for the dermis. Considering that in callus, primary thickening happens via the accumulation of nonviable tissues, metabolic heat generation can be ignored.

Taking into account that we are looking for stationary solutions, Equation (1) for the nonviable (passive) tissue can be reduced to

(3) $$\frac{d^{2}T}{dz^{2}}=0,\;\mathrm{for}\;z=[0,E]$$

with two boundary conditions. At the lower boundary (with the dermis), we can write

(4) $$T{|}_{z=0}=T_{d}$$

Here, *T_d_* is the temperature at the dermal/epidermal interface. At the upper boundary (with air), using Equation (2), we can write

(5) $$-k_{e}{\left.\frac{dT}{dz\;}\right|}_{z=E}=q=h\left(T_{skin}-T_{\infty }\right)$$

Here, *T_skin_* is the temperature at the epidermis/air interface, and *k_e_* is the thermal conductivity of the epidermis (W/m∙K).

The solution to Equation (3) with boundary conditions represented by Equations (4) and (5) is a linear function. Thus, we have a linear temperature drop in the passive layer from *T_d_* (interface with the dermis) to *T_skin_* (interface with the air). However, as the temperature profile within the passive layer is not measurable and has few practical applications, we can use the overall temperature drop across the passive layer (Δ*T_e_*) instead. Thus, we can write

(6) $$T_{d}=T_{skin}+∆T_{e}$$

where

(7) $$∆T_{e}=h\left(T_{skin}-T_{\infty }\right)E/k_{e}$$

In particular, we can explicitly express *T_skin_* through the temperature of the dermal layer and ambient temperature:

(8) $$T_{skin}=\frac{T_{d}+T_{\infty }hE/k_{e}}{1+hE/k_{e}}$$

However, we need to solve an inverse problem for practical purposes: determining the epidermal thickness from skin temperature measurements. 

We know the ambient air temperature $T_{\infty }$ and can measure the surface temperature *T_skin_*; assuming that we know the dermal temperature *T_d_* (e.g., a temperature-stabilized surface such as a blackbody), we can obtain the thickness of the epidermis layer:

(9) $$E=\frac{(T_{d}-T_{skin})k_{e}}{(T_{skin}-T_{\infty })h}$$

However, this expression is not particularly useful in clinical practice, as it contains an unknown dermal temperature, *T_d_*. The problem can be solved by comparing skin temperatures in two (preferably nearby) areas.

Equation (7) for Δ*T_e_* can be rewritten in terms of *T_d_* using Equation (8):

(10) $$∆T_{e}=\frac{(T_{d}-T_{\infty })hE/k_{e}}{1+hE/k_{e}}$$

Now, it has the following structure: $∆T_{e}=(T_{d}-T_{\infty })\tilde{E}/\left(1+\tilde{E}\right)$. Here, $\tilde{E}=hE/k_{e}$ is a dimensionless epidermal thickness. Assuming the stability of the dermal temperature at two nearby points and measuring a temperature difference between these 2 points, we can derive the following:

(11) $$∆T_{12}=-∆T_{e,1}+∆T_{e,2}=(T_{\infty }-T_{d})\left\{\frac{hE_{1}/k_{e}}{1+hE_{1}/k_{e}}-\frac{hE_{2}/k_{e}}{1+hE_{2}/k_{e}}\right\}$$

As *k_e_* = 0.21 W/m/K [14], *h*/*k_e_* can be estimated as 57.1 m^−1^. Thus, *hE*/*k_e_* = 0.0057, 0.086, and 0.11 for the dry, hairy skin, the dry, glabrous epidermis, and the 2 mm callus, respectively.

Taking into account that *hE*/*k_e_* is small in most practical cases, and the denominators in Equation (11) do not drastically differ from 1, we can linearize Equation (11):

(12) $$∆T_{12}=(T_{\infty }-T_{d})\left(E_{1}-E_{2}\right)h/k_{e}$$

Thus, if we take one point (for example, a normal glabrous skin) as a reference point, then the epidermis thickening at another point can be estimated as follows:

(13) $$∆E=\frac{∆T_{12}}{T_{\infty }-T_{d}}k_{e}/h$$

Even though the dermal temperature is unknown, for practical purposes, it can be estimated as the skin temperature or as the skin temperature +0.5–1 °C.

### 2.2. Phantom Experiments

We performed thermographic measurements on inclined gelatin phantoms to validate our predictions in a well-controlled environment.

#### 2.2.1. Phantom Fabrication

The experimental setup begins with preparing the molding material, which will ultimately be molded and transferred onto the platform of a temperature-regulated plate (referred to in the future as the blackbody). The phantom preparation involves using a gelatin and soybean oil emulsion (branded under Intralipid^®^). 

The molding material preparation begins by heating 49 mL of phosphate-buffered saline (PBS) to 80 °C. Seven grams of porcine gelatin was gradually added, and the solution was vigorously stirred until everything was dissolved. The phantom was left to naturally cool at room temperature to 36 degrees Celsius. This process took around 3 min. This was followed by homogeneously mixing 2.8 mL of 4% Intralipid^®^ in with the solution. 

**Figure 1:**
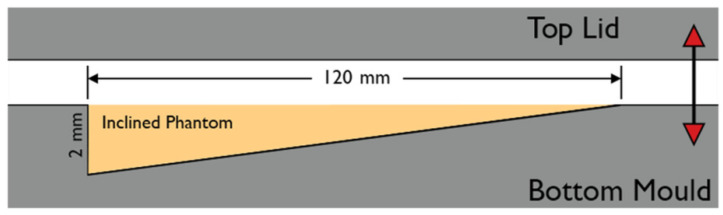
The construction of the inclined phantom.

While still warm, the resulting solution was poured into an aluminum mold. The mold cavity tapered from 2 mm to 0 mm over a distance of 120 mm. The mold was lidded shut with an aluminum plate and left to cool. Figure 1 depicts the construction of the mold.

#### 2.2.2. Thermography Measurements

The lower (thicker) half of the resulting casted body (referred to in the future as the phantom) was cut out and placed onto a transparency sheet so that the phantom would fit into the cavity of the blackbody. Blackbody devices are used to emulate human body temperatures for thermography applications. In the current experiment, we used an SBR-3 (Santa Barbara Infrared Inc., Santa Barbara, CA, USA). The blackbody was capable of maintaining programmed temperatures through an internal control loop. The bottom of the transparency sheet was then thermally coupled to the blackbody stage using water. 

**Figure 2:**
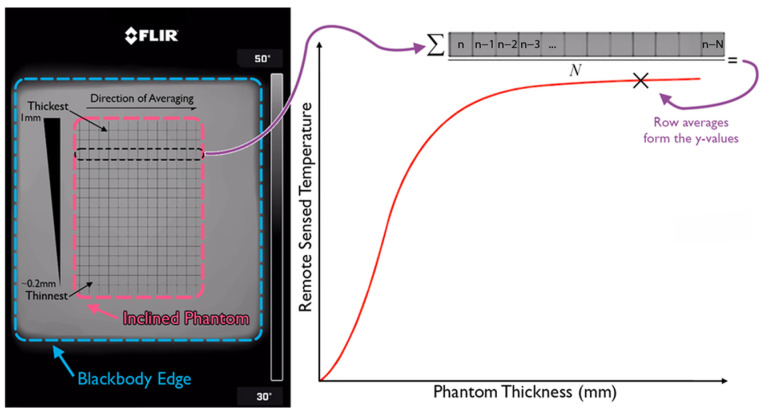
Data analysis flow of the thermographs.

**Figure 3:**
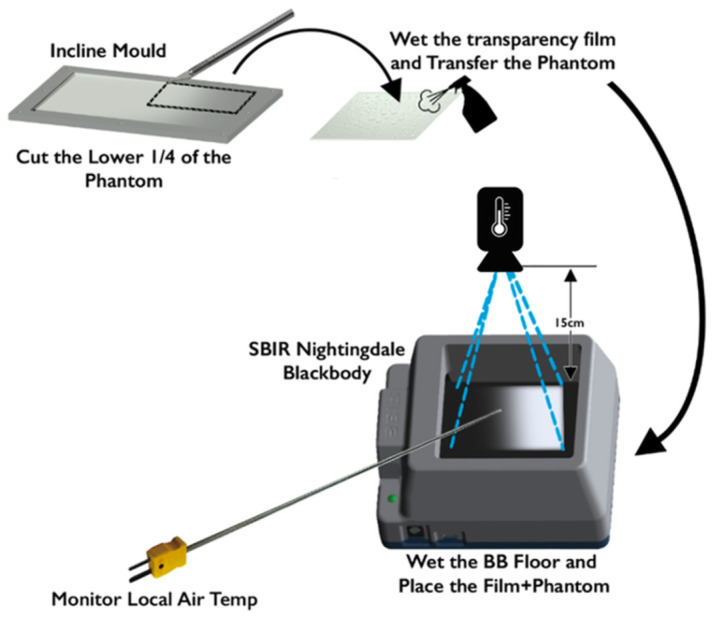
The summary of the experimental setup.

The thermographic measurements were performed using a FLIR One Pro (Teledyne FLIR, Wilsonville, OR, USA) in combination with an iPhone 9 (Apple Inc., Cupertino, CA, USA). The device was positioned 15 cm away from the blackbody stage. Two thermographs were collected, one for a set blackbody temperature of 24 °C and the other for 27 °C. The pixels outlining the phantom were isolated, and a spatial averaging of the pixels was performed alongside the width of the phantom to increase the signal-to-noise ratio, SNR. This kind of operation produced a one-dimensional plot outlining the relationship between the phantom thickness and the apparent temperature in thermography. Figure 2 outlines the flow of the analysis of the thermographs. Figure 3 summarizes the entirety of the experimental materials and methods.

### 2.3. Case Study

The feet of two hospitalized patients referred for debridement were imaged using a Cat S62 smartphone (Caterpillar, Irving, TX, USA) with an integrated FLIR thermal camera. The patients’ extremities were exposed to room air 10 min prior to photography, which included a callous part in the picture at a distance of 10–20 cm. Measures were taken to avoid direct contact with the floor and prevent excessive heat loss.

All patients in the hospital where the study was conducted signed the permission form to use their data “for education and publication purposes”. These statements are part of their medical records. In addition, explicit verbal informed consent was obtained from two case study participants specifically for this paper.

Patient 1:

75-year-old male, post-ICU (Intensive Care Unit) stay due to perforated bowel. Past medical history includes congestive heart failure (CHF), venous insufficiency, palpable pulses, and a perforated bowel ten years ago. 

Patient 2:

71-year-old woman hospitalized due to acute kidney failure. Past medical history includes NIDDM(non-insulin dependent diabetes mellitus), thyroid insufficiency, peripheral vascular diseases, non-palpable pulses, and an ankle brachial index (ABI) of 0.8. The patient was seen for debridement for a diabetic foot ulceration.

## 3. Results

### 3.1. Phantom Experiments

**Figure 4:**
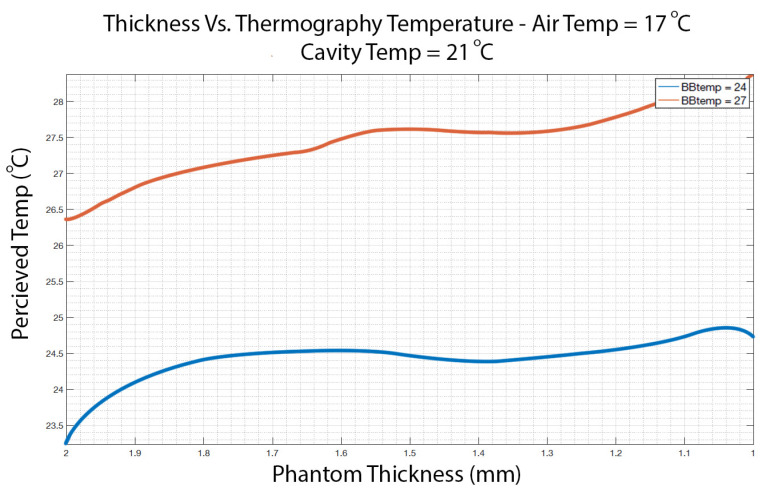
Measured temperature as a function of the phantom thickness. The blackbody temperature was set to 24 °C (blue line) and 27 °C (red line). The ambient temperature was 17 °C, and the cavity temperature was 21 °C.

The results of the phantom temperature measurements are depicted in Figure 4. The ambient temperature was 17 °C, and the cavity temperature was 21 °C. The temperature for each thickness was averaged across multiple pixels with the same thickness (row average, as per Figure 2) to increase the SNR.

From Figure 4, one can see that the increase in phantom thickness by 1 mm (from 1mm on the right to 2 mm on the left) was associated with the decrease in surface temperature by 2 °C and 1–1.5 °C for the black body temperature of 27 °C (red line) and for 24 °C (blue line), respectively.

### 3.2. Case Studies

**Figure 5:**
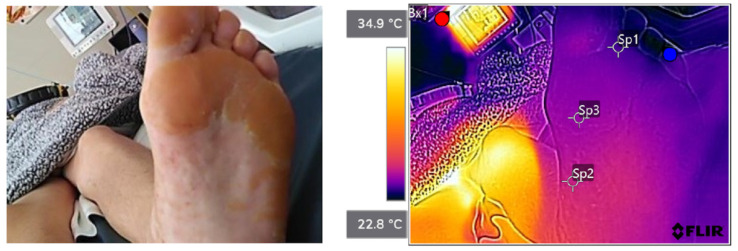
Anatomic (**left pane**) and thermographic (**right pane**) images of the foot with callus. Thickened skin had a brownish color. Temperatures at points 1 and 3 (callus) were 23.9 °C and 24.6 °C, respectively. Point 2 (no callus) had a higher temperature of 25.3 °C. The colormap shows that callus-affected areas had lower temperatures than nearby areas.

Patient 1:

Figure 5 shows anatomic (left pane) and thermographic (right pane) images of the foot with callus. Thickened skin had a brownish color. One can see callous skin on the metatarsal and no callus in the mid-part of the foot. Temperatures at points 1 and 3 (callus) were 23.9 °C and 24.6 °C, respectively. Point 2 (no callus) had a higher temperature of 25.3 °C. 

**Figure 6:**
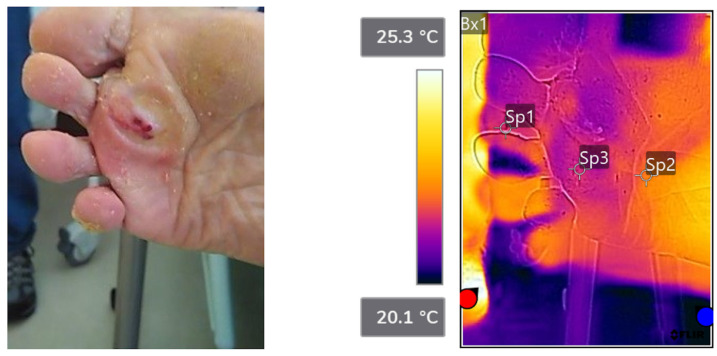
Anatomic (**left pane**) and thermographic (**right pane**) images of the foot with callus. Thickened skin had a brownish color. Temperatures at points 1 and 3 (callus) were 21.8 °C and 21.7 °C, respectively. Point 2 (no callus) had a higher temperature of 22.4 °C. The colormap shows that callus-affected areas had lower temperatures than nearby areas.

Patient 2:

Figure 6 shows anatomic (left pane) and thermographic (right pane) images of the foot with callus. Thickened skin had a brownish color. Temperatures at points 1 and 3 (callus) were 21.8 °C and 21.7 °C, respectively. Point 2 (no callus) had a higher temperature of 22.4 °C. 

## 4. Discussion

Our analytical calculations, phantom experiments, and case studies demonstrate the feasibility of using thermography for callus detection.

The analytical model (see Equation (12)) shows that, all else being equal, the glabrous skin, due to its larger epidermis thickness (up to 1.5 mm, [15]), is $0.08(T_{\infty }-T_{d})$ degrees cooler than nearby non-glabrous skin. In realistic healthcare settings, it can be on the order of 0.8 °C.

Similarly, a further 1 mm increase in the epidermis thickness of the glabrous skin (2.5mm in total) will be $0.048(T_{\infty }-T_{d})$ degrees cooler than nearby glabrous skin. In realistic healthcare settings, it can be on the order of 0.5°C.

Our theoretical predictions were confirmed in phantom and clinical case studies on two patients (with and without a diabetic foot ulcer). The thermographic imaging of plantar feet revealed that areas with callus have temperatures at least 0.6–0.7 °C lower than those of nearby callus-free areas. Moreover, from colormaps, one can see that callus-affected areas have lower temperatures than nearby areas; thermal imaging can thus be potentially used to delineate the callus-affected areas.

As such, our results confirm the notion introduced in [11] that thermography can be used for callus identification. Thus, it further pushes the boundaries for the clinical use of thermography, particularly in wound care. 

The clinical implications of proper callus identification could be vast. Firstly, patients can use it as part of the daily assessments that practitioners usually recommend. For example, when they measure temperature and see it going down, they can be alerted that a professional assessment is needed. Furthermore, this alert can also be sent to the treating practitioner. This system would be very similar to those used by people with diabetes who manage their blood sugar levels (i.e., when their blood sugars increase, an alert is sent both to the patient and to the practitioner). Secondly, novice practitioners can evaluate whether or not adequate debridement of the area was achieved. Finally, thermographic imaging can be used for documentation purposes to justify the selection and size of the debridement area.

The utility of callous area identification can be extended from wound care to foot care in general. Antończak et al. [16] found that podiatry treatments consisting of the removal of hyperkeratosis not only normalized the parameters related to foot geometry and to forefoot pressure on the ground, but also reduced foot pain, thus improving quality of life.

Treatment of hyperkeratosis is dependent on its severity. According to the American Podiatric Medical Association, mild corns and calluses may not require treatment. Larger corns and calluses are most effectively reduced with a surgical blade [17].

However, current callus identification and staging options are limited to visual inspection. Medical professionals, such as dermatologists or podiatrists, can visually assess and identify calluses based on their appearance, location, and texture. Thermography can help with the identification of callous areas and their staging. Moreover, routine use of thermography could help identify other serious foot conditions. For example, skin temperature variations of over 2.0 °C could be helpful for identifying pathological conditions in the diabetic foot [18].

Thermographic imaging has multiple advantages. For example, with the advances in thermographic sensors, they have become quite affordable, which makes them suitable for routine use in many clinical scenarios. In addition, thermography is a passive modality, as it measures energy flow from the object. As such, it does not pose any risk to patients. 

It should be noted that certain measures need to be taken to obtain thermographic images that have diagnostic potential [19]. For example, Villar Rodriguez et al. [20] suggested keeping patients’ feet uncovered for 5 min to avoid the influence of footwear type and the state of their feet on arrival and to adapt to ambient conditions. Moreover, measures must be taken to avoid direct contact with the floor and to prevent excessive heat loss [20].

While adopting the proposed approach, certain considerations need to be taken into account. Firstly, while thermographic imaging works for any type of object, the proposed effect will be observed in vivo only as a function of the temperature gradient.

Secondly, our analytical calculations show the importance of ambient temperature measurements during thermographic assessments. In particular, Equation (13) contains the $T_{\infty }-T_{d}$ scaling factor. Thus, the effect’s magnitude will significantly differ for ambient temperatures of 20 °C or 24 °C. For example, assuming an ambient temperature of 24 °C and a normal skin temperature of 34 °C, a local temperature decrease to 33.5 °C can be associated with epidermal thickening by an additional 0.9mm. The same skin temperature readings for an ambient temperature of 20 °C would correspond to 0.6 mm thickening.

Thirdly, we considered a simplified mechanism. Callus, particularly in diabetic foot ulcer cases, can have a much more complex impact on skin temperature. In particular, a thick callus may significantly impact the skin’s elasticity; thus, microcirculation can be negatively impacted, thus lowering the temperature of the skin even further. In another hypothetical scenario, callus may cover an inflammation site. As inflammation is typically associated with elevated temperatures, certain callus areas over inflammation sites may have higher temperatures than nearby callus-free areas. Thus, further research is required.

The work itself has certain limitations. 

Gelatin phantoms are valid skin models for tissue optics. However, their utility in thermographic studies is yet to be established. In particular, while the thermal conductivity of gel phantoms is close to that of the skin [21], it depends significantly on the water content [22]. As the phantom dries out, its thermal conductivity could change significantly. The same applies to the epidermis itself. Heat transfer depends significantly on the thermal conductivity of the epidermis, which depends on the water content. Thus, it is essential to understand the thermophysical properties of the stratum corneum of a particular body part. The thermal properties of the stratum corneum across several body locations were measured in [23]. In particular, the authors found a significant dissimilarity in the callus-prone areas of the palm and heel in terms of stratum corneum thickness (260 vs. 1100 μm), water content (45 vs. 10%), and thermal conductivity (0.36 vs. 0.28 W/m/K).

Due to the geometry of the blackbody cavity (see Figure 3), the ambient temperature for the phantom was difficult to gauge. To address this uncertainty, we included measurements for ambient temperature in the room and for air temperature in the cavity.

In addition, our clinical studies were limited to two patients. A small number of patients does not allow generalization of the results. However, clinical validation was not in the scope of the current study. We presented clinical data to demonstrate feasibility only. 

In future work, the corresponding clinical validation will be conducted on a larger patient population.

## 5. Conclusions

We performed a theoretical analysis, validated our predictions on gelatin phantoms, and demonstrated the feasibility of thermographic imaging for callus identification in two clinical case series. Our experimental results are in agreement with theoretical predictions and support the notion that local skin temperature variations can indicate epidermis thickness variations, which can be used for callus identification. In particular, a surface temperature drop on the order of 0.5 °C or more can be indicative of callus presence, particularly in callus-prone areas. In addition, our analytical calculations and phantom experiments show the importance of ambient temperature measurements during thermographic assessments.

## Data Availability

No new data were created or analyzed in this study. Data sharing is not applicable to this article.
